# Supplementary material for: Respiratory Syncytial Virus (RSV) Disease and Prevention Products: Knowledge, Attitudes, and Preferences of Kenyan Healthcare Workers in Two Counties in 2021
Source: Vaccines (Basel). 2023 Jun 2;11(6):1055. doi: 10.3390/vaccines11061055 (PMC10302044; doi:10.3390/vaccines11061055)
Supplement: Supplementary file 1 [file vaccines-11-01055-s001.zip › vaccines-2392756-supplementary.pdf]

Sup Table S1: Healthcare workers (HCWs) awareness of vaccines delivered in pregnancy in Siaya and Nairobi Counties, Kenya.

Characteristic	Overall, N=106	Siaya, N=60	Nairobi, N=46	p-value
	n	n (%)	n	
Aware of vaccines available for pregnant women				
Yes	105 (99.0)	59 (98.3)	46 (100)	0.99
Vaccines that HCWs are aware of				
Tetanus Toxoid vaccine	104 (99.0)	58 (98.3)	46 (100)	0.99
Diphtheria vaccine	32 (30.5)	13 (22.0)	19 (41.3)	0.03
Influenza vaccine	13 (12.4)	6 (10.2)	7 (15.2)	0.44
Pertussis vaccine	1 (1.0)	1 (1.7)	0 (0)	0.99
Human Papilloma Virus Vaccine	4 (3.8)	3 (5.1)	1 (2.2)	0.63
COVID-19	17 (16.2)	4 (6.8)	13 (28.3)	0.01
Other	12 (11.4)	6 (10.2)	6 (13.0)	0.65
Vaccines that HCWs recommend for pregnant women				
Tetanus Toxoid vaccine	80 (76.2)	42 (71.2)	38 (82.6)	0.17
COVID-19	39 (37.1)	15 (25.4)	24 (52.2)	0.01
Malaria	22 (21.0)	20 (33.9)	2 (4.4)	<0.01
Reason why recommend vaccine				
To protect the mother	93 (88.6)	47 (79.7)	46 (100)	<0.01
To protect the baby	78 (74.3)	43 (72.9)	35 (76.1)	0.71
Required by government	8 (7.6)	2 (3.4)	6 (13.0)	0.13
Other	24 (23.3)	16 (27.1)	8 (17.4)	0.24
Malaria endemic zone	9/24 (37.5)	9/16 (56.3)	0/8 (0)	
Aware of alternative technologies such as monoclonal antibodies currently available for pregnant women or their infants	14 (13.2)	8 (13.3)	6 (13.0)	0.97
Perceived gestational age at which to vaccinate for optimal effectiveness				
<16 weeks	43 (40.6)	27 (45.0)	16 (34.8)	0.29
16-32 weeks	63 (59.4)	33 (55.0)	30 (65.2)	
With respect to vaccination schedule, which schedule would pregnant women prefer?				
Single dose	79 (74.5)	45 (75.0)	34 (73.9)	0.90
Multiple dose	27 (25.5)	15 (25.0)	12 (26.1)	
With respect to vaccination schedule, which schedule would you recommend for pregnant women?				
Single dose	62 (58.5)	37 (61.7)	25 (54.4)	0.45
Multiple dose	44 (41.5)	23 (38.3)	21 (45.7)	
Considering vaccines presented in single dose vials/ devices or multi-dose vials, which one would you recommend for maternal immunization? (n=86)				
Single dose	50 (58.1)	27 (58.7)	23 (57.5)	0.92
Multiple dose	36 (41.9)	19 (41.3)	17 (42.5)	
Are you concerned about adverse birth outcomes following maternal immunization?				
Yes	57 (54.8)	39 (65.0)	18 (39.1)	0.01
In your opinion, which maternal immunization strategy would be the most feasible?				
Antenatal care clinics	53 (50.0)	27 (45.0)	26 (56.5)	0.03
Mass campaigns	23 (21.7)	10 (16.7)	13 (28.3)	
Other (Mobile outreaches)	30 (28.3)	23 (38.3)	7 (15.2)	
In your opinion, which maternal immunization strategy would be the most effective?				
Antenatal care clinics	53 (50.0)	26 (43.3)	27 (58.7)	0.04
Mass campaigns	26 (24.5)	13 (21.7)	13 (28.3)	
Other (Mobile outreaches)	27 (25.5)	21 (35.0)	6 (13.0)	
Have you heard about respiratory syncytial virus (RSV)*				
Yes	41 (39.4)	25 (41.7)	16 (36.4)	0.58

*Only applies for the non-KENITAG participants.
